# Diagnostic accuracy of pre-hospital invasive arterial blood pressure monitoring for haemodynamic management in traumatic brain injury and spontaneous intracranial haemorrhage

**DOI:** 10.1186/s13049-025-01393-4

**Published:** 2025-05-16

**Authors:** J. E. Griggs, S. Clarke, R. Greenhalgh, A. N. Watts, J. Barrett, S. Houghton Budd, M. Dias, K. Hunter, R. M. Lyon, E. ter Avest, On behalf of Air Ambulance Charity Kent Surrey Sussex

**Affiliations:** 1 Air Ambulance Kent Surrey Sussex, Redhill Aerodrome, Hanger 10, Redhill, Surrey RH1 5YP UK; 2 Faculty of Health Sciences, University of Surrey, Guildford, GU2 7XH UK; 3 Royal Sussex Country Hospital, Brighton, UK; 4 South East Coast Ambulance Foundation Trust, Crawley, UK; 5 Department of Acute Care, University Medical Center Groningen, Groningen, The Netherlands; 6 London’s Air Ambulance, London, UK

**Keywords:** Pre-hospital emergency medicine, Traumatic brain injury, Spontaneous intracranial haemorrhage, Invasive arterial blood pressure, Non-invasive blood pressure, Neuroprotection, Bland-Altman Analysis, Error Grid Analysis

## Abstract

**Background:**

Neuroprotective measures to prevent secondary brain injury are a critical aspect of pre-hospital management in patients with acute traumatic brain injury (TBI) and spontaneous intracranial haemorrhage (sICH). Haemodynamic optimisation guided by non-invasive blood pressure (NIBP) measurements is an important neuroprotective measure, as cerebral autoregulation is often absent or impaired. The accuracy and clinical relevance of invasive arterial blood pressure (IBP) monitoring to optimise haemodynamic management has not been established in patients with a brain insult.

**Methods:**

A retrospective clinical diagnostic accuracy study to establish the accuracy and clinical relevance of IBP-guided haemodynamic optimisation in patients with TBI or sICH. The occurrence- and clinical relevance of IBP-NIBP discrepancies in patients attended by a UK Helicopter Emergency Medical Service (HEMS) between 6 January 2022 and 6 January 2024 was evaluated. Bland-Altman plots with adjustment for repeated measures were constructed to analyse *disagreement* in relation to absolute blood pressure values. Multivariate analysis was performed using generalised linear mixed effects regression (GLMER) models with random effects to identify predictors of disagreement. Error Grid Analysis (EGA) classified the clinical relevance of discrepancies. The primary outcome was *pairwise agreement* between IBP and NIBP, defined as less than 10% difference in mean arterial pressure (MAP).

**Results:**

For 209 patients (159 TBI and 50 sICH) 1020 concurrent IBP and NIBP measurements were available. The average [95% CI] difference in MAP was -1.4 mmHg (-3.09 to 0.27) and 2.6mmHg in TBI. Only 459 (54.7%) MAP data met criteria for pairwise agreement. Multivariate regression analysis revealed a strong association between MAP disagreement and ground emergency medical service conveyance (aOR 2.01, 95% CI 0.98-4.10). Bland-Altman analysis demonstrated proportional bias, with NIBP *under*estimation of MAP at higher blood pressures and *over*estimation at lower blood pressures. EGA revealed that in 6.1% (95% CI: 4.5-7.7) of TBI and 12.5% (95% CI: 7.8-17.2) of patients with sICH pairwise disagreement was associated with a moderate to dangerous risk of over- or undertreatment.

**Conclusion:**

NIBP guided pre-hospital haemodynamic management of patients with TBI or sICH is hampered by clinically relevant measurement inaccuracies in a significant proportion of patients. Pre-hospital IBP has the potential to improve early haemodynamic optimisation, especially when hypo- or hypertension is present, enabling tailored neuroprotection in the hyperacute phase.

**Supplementary Information:**

The online version contains supplementary material available at 10.1186/s13049-025-01393-4.

## Background

Neuroprotective measures to prevent secondary brain injury are a critical aspect of pre-hospital management in patients with acute traumatic brain injury (TBI) and spontaneous intracranial haemorrhage (sICH) [1, 2]. Haemodynamic optimisation is an important neuroprotective measure in TBI patients. Cerebral autoregulation is absent or impaired in 49–87% of these patients [1], therefore maintaining appropriate mean arterial pressure (MAP) is crucial to preserve adequate cerebral blood flow (CBF) and prevent secondary brain injury [3–5].

Pre-hospital critical care teams such as helicopter emergency medical services (HEMS) have the capability to provide both volume replacement and vasopressor therapy guided by non-invasive blood pressure measurements (NIBP) [2]. However, NIBP measurements can be confounded by movement artefact, and vibratory effects during transport, and may be less reliable in patients with severe *hypo* and *hyper*tension [6–10]. This may result in artefactual or intermittent measurements which either *over-* or *under*estimate blood pressure values [3–5], with important consequences for subsequent initiation- or withholding of treatment [11] over extended conveyance intervals [6].

Invasive arterial blood pressure (IBP) monitoring may offer a more reliable alternative [5, 7]. Retrospective studies have demonstrated the feasibility and efficiency of IBP in pre-hospital critical care [8–10]. Additionally, a recent study reported higher accuracy of IBP compared to NIBP in a heterogeneous cohort of pre-hospital patients [5]. However, two important questions remain unclear. First, the degree of inaccuracy in non-invasive measurements compared to invasive measurements. Second, whether such measurement differences translate to clinically relevant advantages for haemodynamic optimisation in patients with a brain insult.

This study aims to establish the accuracy and clinical relevance of IBP-guided haemodynamic optimisation in patients with TBI or sICH and determine if there is a perceived clinical advantage to guide neuroprotective measures in the pre-hospital phase.

## Methods

### Study setting and design

A retrospective observational diagnostic accuracy study was performed using data extracted from our pre-hospital electronic patient clinical record database (HEMSBase™ 3.0, MedicOne Systems). We analysed data from patients attended by HEMS in South-East England between January 6, 2022, and January 6, 2024. The data was collected as part of routine clinical care and retrospectively analysed for this study. Ambulance Charity Kent Surrey Sussex (KSS) provides pre-hospital advanced interventions within the associated major trauma systems in conjunction with ground emergency medical services (EMS). KSS operates across three counties serving a resident population of 5 million. A physician-paramedic pre-hospital emergency medicine team respond by either helicopter or rapid response vehicle. Doctors have a minimum of 5-years postgraduate experience, including a minimum of 6-months anaesthesia training. Paramedics undergo further specialist practice, including theoretical modules on invasive monitoring and pre-hospital emergency anaesthesia (PHEA). Subsequently, clinicians demonstrating clinical competency may then site pre-hospital invasive monitoring. Advanced interventions (including transfusion of blood components, airway management, anticoagulant reversal and surgical procedures) are delivered during the pre-hospital phase both at scene and during conveyance [12]. IBP monitoring was introduced in January 2022 in a governed stepwise manner following expert consensus by consultants and specialists in pre-hospital emergency medicine.

### Patient selection

Patients were eligible for inclusion if they were 18 years or older and were attended by KSS between 6 January 2022 and 6 January 2024 for suspected TBI or sICH. Only patients in whom an arterial cannula was successfully sited and for whom concurrent IBP and NIBP measurements available were included. Paediatric patients and interhospital transfers were excluded from the present analysis [13].

### Non-invasive and invasive arterial blood pressure monitoring

Non-invasive blood pressure measurements were performed using appropriately sized cuffs based on patient arm circumference, following manufacturer recommendations. The NIBP cuff is placed on the upper arm contralateral to the arterial line when possible. Measurements are taken at 3-min intervals unless manually triggered when clinically indicated.

According to KSS Standard Operating Procedure (SOP) invasive arterial blood pressure monitoring is indicated for patients with suspected TBI and sICH where HEMS clinicians perceive continuous invasive BP monitoring will contribute to haemodynamic optimisation. Arterial cannulation (Vygon SWITCH, 20 g, 45 mm length, Utah, US; Arterial Leadercath, 4 F, 10 cm Length, Vygon, France; or Ultimum^TM^ Hemostasis Introducer, 5 F, 12 cm length, Abbott Medical, USA) is commonly performed prior to PHEA, with the radial artery being the preferred access. The chosen device is connected to a disposable pressure monitoring set (TruWave^TM^, PX260, Edwards Lifesciences, Irvine, CA, USA) via a 150-cm-long saline line with a pressure infusion bag (ACCU-PRO^TM^, APPI00500, 500 ml, ProAct Medical Ltd, Northants, UK). The transducer is placed at the level of the right atrium. Both invasive- and non-invasive blood pressures are measured using the Tempus PRO^TM^ (Phillips Electronics, Farnborough, UK) device with arterial waveforms visible to clinicians in real-time. Data capture is at 3-min intervals for non-invasive and 1-min intervals for invasive monitoring.

Clinicians were trained to identify and address waveform artefacts, particularly issues related to overdamping and underdamping. Overdamping artefacts may include systolic blood pressure (SBP) underestimation, diastolic blood pressure (DBP) overestimation, slurred upstroke, absent dicrotic notch, and general loss of waveform detail. These were addressed by checking for underinflated pressure bags, air bubbles, blood clots, loose connections, and catheter kinking. Underdamping artefacts may include: SBP overestimation, DBP underestimation, exaggerated dicrotic notch, and non-physiological oscillations) and were addressed by examining tubing stiffness and transducer function.

### Primary and secondary outcomes

The primary outcome was the percentage of concurrent blood pressure measurements demonstrating *pairwise agreement* between IBP and NIBP, defined as a <10 mmHg (MAP), or <20 mmHg (SBP and DBP) difference. Concurrent data pairs refer to one invasive and one non-invasive measurement recorded from the same patient within a 1-minute interval on the electronic patient clinical record (EPCR).

Secondary outcomes were:

1. *Pairwise agreement* using alternative definitions (<20% or <30% difference for MAP and <30% or <40% difference for SBP/DBP).
2. Patient- and/or treatment characteristics independently associated with pairwise *disagreement*.
3. The presence- (and magnitude) of a relation between absolute blood pressure values and the magnitude of IBP-NIBP discrepancies (proportional bias).
4. The estimated clinical relevance of any pairwise disagreement present, as quantified by Error Grid Analysis (EGA).

### Data sources and data acquisition

Data was extracted through Zoho Analytics^TM^ from the EPCR HEMSBase^TM^ 3.0 (MedicOne Systems) and included the following categories. Baseline demographic descriptors: age, sex and weight (clinician estimated). Physiological parameters included: Glasgow Coma Scale (GCS) score, Heart rate (HR), Oxygen saturation (SpO_2_), End-tidal Carbon dioxide (EtCO_2_), SBP, invasive SBP (iSBP), DBP, invasive DBP (iDBP), MAP and invasive MAP (iMAP). Pre-hospital advanced interventions: Arterial line catheterisation time and site [radial, brachial, femoral], type of arterial catheter used [Flowswitch, Leadercath], blood component transfusion [yes/no], vasoactive drug administration [yes/no].

Blood pressure measurements were screened for artefacts using a two-stage process. First, predefined thresholds were applied to exclude physiologically implausible measurements based on criteria from Juri et al. [14]: invasive SBP >300 mmHg or <20 mmHg, invasive DBP >225 mmHg or <5 mmHg, and pulse pressure (difference between iSBP and iDBP) <5 mmHg. These exclusion criteria were applied during data pre-processing. Second, the EPCR were reviewed by a panel of clinicians to identify artefactual invasive and non-invasive measurements, these pertained to measurements perceived to be physiologically implausible i.e. did not fit the physiological trend of the data.

### Ethical considerations and data governance

Data were routinely collected and met Health Research Authority (HRA, UK) criteria for service evaluation. Research Ethics Committee approval was not required, and the project was approved by the KSS Research and Innovation Committee. Study conduct was in accordance with Strengthening the Reporting of Observational Studies in Epidemiology (STROBE) [15].

### Statistical analysis

Baseline characteristics (stratified for brain injury aetiology) were compared across groups using Chi Square (*X*^2^) test for categorical data and Mann-Whitney U test for continuous data. Mean differences (SD) between NIBP and IBP were calculated, and Bland-Altman analysis with adjustment for repeated measurements [16, 17] was performed to investigate the relation of mean differences with absolute blood pressure values. Mean differences were related to pre-defined limits of agreement between NIBP and IBP. Univariate regression analysis was performed using a generalised linear mixed model (GLMM) with binomial logit to identify variables related to disagreement at the pre-defined 10% acceptability threshold. Multivariate regression was performed with those factors that showed an *R*^*2*^ >0.20 in univariate analysis to investigate which variables were independent predictors of MAP disagreement.

Error grid analysis was performed to establish clinical relevance. Differences between invasive and non-invasive blood pressure measurements were classified into five risk levels, ranging from zone A (no risk, no difference in clinical action) to zone E (dangerous risk, unnecessary (absence of) treatment with severe life-threatening consequences) [18]. Risk zones were calibrated based on the aggregated results of a survey amongst 25 specialists in intensive care medicine and anaesthesiology) [18].

Data were pre-processed in Microsoft Excel (Microsoft Corp, Redmond, WA USA). Statistical analysis and visualisations were performed using R version 4.3 (R Core Team, 2023. R: A language and Environment for Statistical Computing. R Foundation for Statistical Computing, Vienna, Austria). R packages included: *base r* stats for logistic regression and model comparison, *blandr* for Bland-Altman Analysis, *ggplot2* and *flextable* for creating plots and table creation, *dplyr* for data manipulation, *broom* for tidying model outputs, *lme4* for mixed effects models. EGA was performed in Matlab 2019 a/b (The Mathworks Inc, Natwick, MA) [18]. Statistical significance was pre-determined at p <0.05.

## Results

### Baseline characteristics

**Fig. 1:**
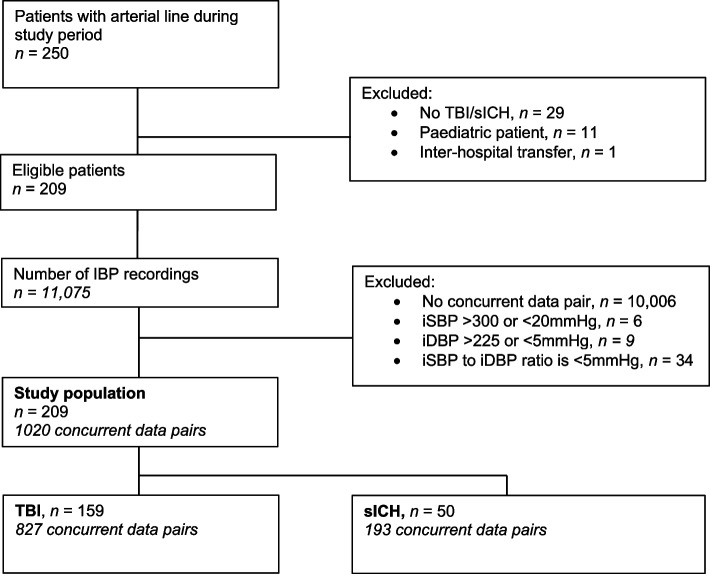
Consort diagram for derivation of study population and frequency of concurrent data pairs. Legend. Derivation of study population and frequency of concurrent data pairs. IBP; invasive arterial blood pressure; EPCR, electronic patient clinical record. TBI; traumatic brain injury. sICH; spontaneous intracranial haemorrhage. iSBP; Invasive systolic blood pressure. iDBP; Invasive diastolic blood pressure

During the study period, an arterial line was sited in 250/3840 patients attended by the service, 221 of which had a suspected TBI or sICH. A total of 209 were eligible for inclusion (Fig. 1). For these patients, a total of 1020 IBP-NIBP concurrent data pairs were available for comparison (median number of measurements per patient was 9 (IQR 3–15, range: 1–29).

Baseline characteristics of the study population (stratified by aetiology of brain injury) are presented in Table 1. Most patients were male (185/209, 77%), with a median age of 51.2 [40–68] years. The radial artery was the preferred site for arterial catheterisation, and over 80% of the arterial lines were placed during the first attempt. In those patients whom received PHEA (198/209, 94.7%) an arterial line was sited pre-PHEA in 153/198 (77.3%). Vasopressors were used in 73 (44.5%) patients, and blood components were administered in 12 (5.8%) patients. Differences between invasive- and non-invasive *average* DBP, MAP and SBP are reported (Table 1). Average difference for TBI was 2.6 mmHg. Further, the mean absolute differences in MAP between non-invasive and IBP are represented in Table 2.

**Table 1:** Baseline characteristics, physiological parameters and clinical interventions of patients with concurrent IBP and NIBP measurements, stratified by aetiology of brain injury (n = 209)

**Variables**	**Overall** **(***n***=209)**	**TBI** **(***n***=159)**	**sICH** **(***n***=50)**	***p-value***
**Demographics**				
Age, median [IQR]	51.2 [40–68]	57 [34–69]	45 [41–55]	0.403
Sex, male [n, %]	185 [77.1]	125 [79.0]	60 [76.0]	0.536
Estimated weight, mean [SD]	76.2 [15.2]	75.3 [15.4]	79.0 [14.4]	0.119
**Baseline Physiological parameters at presentation**				
Presenting GCS, median [IQR]	6 [4–8]	6 [3–9]	6 [4–8]	0.12
HR, median [IQR]	93 [79–106]	93 [80–106]	95 [75–111]	0.426
SpO_2_, median [IQR]	99 [97–100]	99 [97–100]	98 [95–100]	<0.018*
SBP, median [IQR]	128 [112–150]	130 [113–152]	120 [101–139]	<0.001***
iSBP, median [IQR]	135 [113–159]	138 [118–162]	120 [104–142]	<0.001***
DBP, median [IQR]	83 [72–96]	84 [74–96]	79 [65–93]	0.036*
iDBP, median [IQR]	75 [65–88]	76 [66–89]	73 [62–85]	0.002**
MAP, median [IQR]	98 [86–113]	99 [88–114]	93 [78–110]	0.005**
iMAP, median [IQR]	96 [83–114]	99 [85–117]	89 [76–103]	<0.001***
EtCO_2_, median [IQR]	4.3 [3.9–4.8]	4.3 [3.9–4.7]	4.7 [4.0–5.2]	<0.001***
**Catheterisation Timing**				
Pre-PHEA [n, %]	153/198 [77.3]	116/156 [74.4]	37/42 [88.1]	0.059
*Missing data*	19 [0.2]	13 [.06]	6 [.14]	
**Catheterisation Site**				
Radial [n, %]	154 [73.3]	113 [75.3]	41 [91.1]	<0.001***
Brachial [n, %]	4 [1.9]	3 [2.0]	1 [2.2]	
Femoral [n, %]	8 [3.8]	5 [3.3]	3 [6.7]	
*Missing data*	43 [20.5]	38 [23.8]	5 [10]	
**Catheterisation Device**				0.200
Flow switch [n, %]	148 [70.8]	116 [73.0]	32 [64.0]	
Leadercath Vygon [n, %]	52 [24.9]	35 [22.0]	17 [34.0]	
5 FR Ultimum [n, %]	2 [1.0]	1 [0.6]	1 [2.0]	
*Missing data*	7 [4.4]	0 [0]	0 [0]	
**Attempts**				0.920
1 [n, %]	161 [80.5]	120 [80.0]	41 [82.0]	
2 [n, %]	34 [17.0]	26 [17.3]	8 [16.0]	
3 [n, %]	5 [2.5]	4 [2.7]	1 [2.0]	
*Missing data*	9 [4.3]	0 [0]	0 [0]	
**HEMS Interventions**				
PHEA [n, %]	198 [94.7]	156 [98.1]	42 [84.0]	<0.001***
Blood component transfusion^a^, [n, %]	12 [5.8]	12 [7.6]	0 [0.0]	0.048*
Vasopressor use [n, %]	73 [44.5]	58 [36.5]	15 [62.5]	0.015*
**Conveyance**				0.557
Aircraft [n, %]	89 [42.6]	70 [44.0]	19 [38.0]	
Ground EMS [n, %]	120 [57.4]	89 [56.0]	31 [62.0]	

*GCS* Glasgow Coma Score; *HR* heart rate; *SpO*_*2*_ oxygen saturation; *SBP* systolic blood pressure; *DBP* diastolic blood pressure; *EtCO*_*2*_ End-tidal CO_2_.; *MAP* mean arterial pressure; *IQR* interquartile range; *PHEA* pre-hospital emergency anaesthesia; *EMS* emergency medical service

^a^blood component transfused was fresh-frozen or freeze-dried plasma. Statistical significance is denoted by *** for *p* < 0.001, ** for *p* < 0.01, *for *p* < 0.05

**Table 2:** Mean absolute difference (95% CI) between invasive- and non-invasive blood pressure

**Blood Pressure Measurement**	**Mean absolute difference (95% CI)**		
	**Overall** **(***n***=209)**	**TBI** **(***n***=159)**	**sICH** **(***n***=50)**
Abs (MAP – iMAP)	16.0 (14.7–17.4)	15.1 (11.7–18.5)	17.4 (13.9–21.0)
Abs (SBP – iSBP)	21.6 (20.2–22.9)	23.0 (18.6–27.5)	22.8 (19.1–26.5)
Abs (DBP – iDBP)	18.7 (17.2–20.2)	16.2 (9.3–23.1)	19.2 (15.5–22.9)

*Abs* mean absolute difference; *MAP* mean arterial pressure; *iMAP* invasive mean arterial pressure; *SBP* systolic blood pressure; *iSBP* invasive systolic blood pressure; *DBP* diastolic blood pressure; *iDBP* invasive diastolic blood pressure**;** *TBI* traumatic brain injury; *sICH* spontaneous intracranial haemorrhage

Using the most conservative acceptability threshold for pairwise agreement between IBP and NIBP, (<10% difference for MAP and <20% for SBP and DBP), 57.4% of the data pairs had a difference below the threshold for MAP, 77.0% for SBP and 65.5% for DBP. As expected, agreement increased with lower thresholds (higher acceptable disagreement), but even at a disagreement threshold of 30% for MAP, in 12.9% of all measurements the threshold was not met. SBP agreement was consistently higher then DBP agreement (Table 3).

**Table 3:** Pairwise agreement (%) for mean arterial pressure, systolic blood pressure and diastolic blood pressure between invasive and non-invasive measurements in patients with TBI and sICH

**Blood pressure Measurement**	**Acceptability** **Threshold**	**Overall % (95% CI)**	**TBI % (95% CI)**	**sICH % (95% CI)**
Mean arterial pressure	≤10%	56.4 (53.3–59.4)	57.4 (54.0–60.8)	51.8 (44.7–58.9)
	≤20%	79.3 (76.7–81.7)	80.9 (78.2–83.6)*	72.5 (66.1–78.9)
	≤30%	86.2 (83.8–88.2)	87.1 (84.8–89.4)	82.4 (77.0–87.8)
Systolic blood pressure	≤20%	76.1 (73.3–78.6)	77.0 (74.1–79.9)	72.0 (65.6–78.4)
	≤30%	87.1 (84.8–89.0)	88.3 (86.1–90.5)*	81.9 (76.4–87.4)
	≤40%	92.5 (90.7–94.0)	93.2 (91.4–95.0)	89.6 (85.3–93.9)
Diastolic blood pressure	≤20%	64.9 (61.9–67.8)	65.5 (62.2–68.8)	62.2 (55.3–69.1)
	≤30%	77.7 (75.0–80.2)	79.2 (76.4–82.0)*	71.5 (65.0–78.0)
	≤40%	84.8 (82.4–86.9)	86.1 (83.7–88.5)*	79.3 (73.5–85.1)

Acceptability was defined as percentage of pairwise agreement in concurrent measurements, at ≤10/≤20/≤30 mmHg for MAP and ≤20/≤30/≤40 mmHg for SBP and DBP. Analysis based on 1020 paired measurements; TBI, traumatic brain injury; sICH; spontaneous intracranial haemorrhage. Chi-square tests were used to compare agreement percentages between TBI and sICH. Statistical significance is denoted by * for p <0.05

### Factors associated with pairwise disagreement

**Fig. 2:**
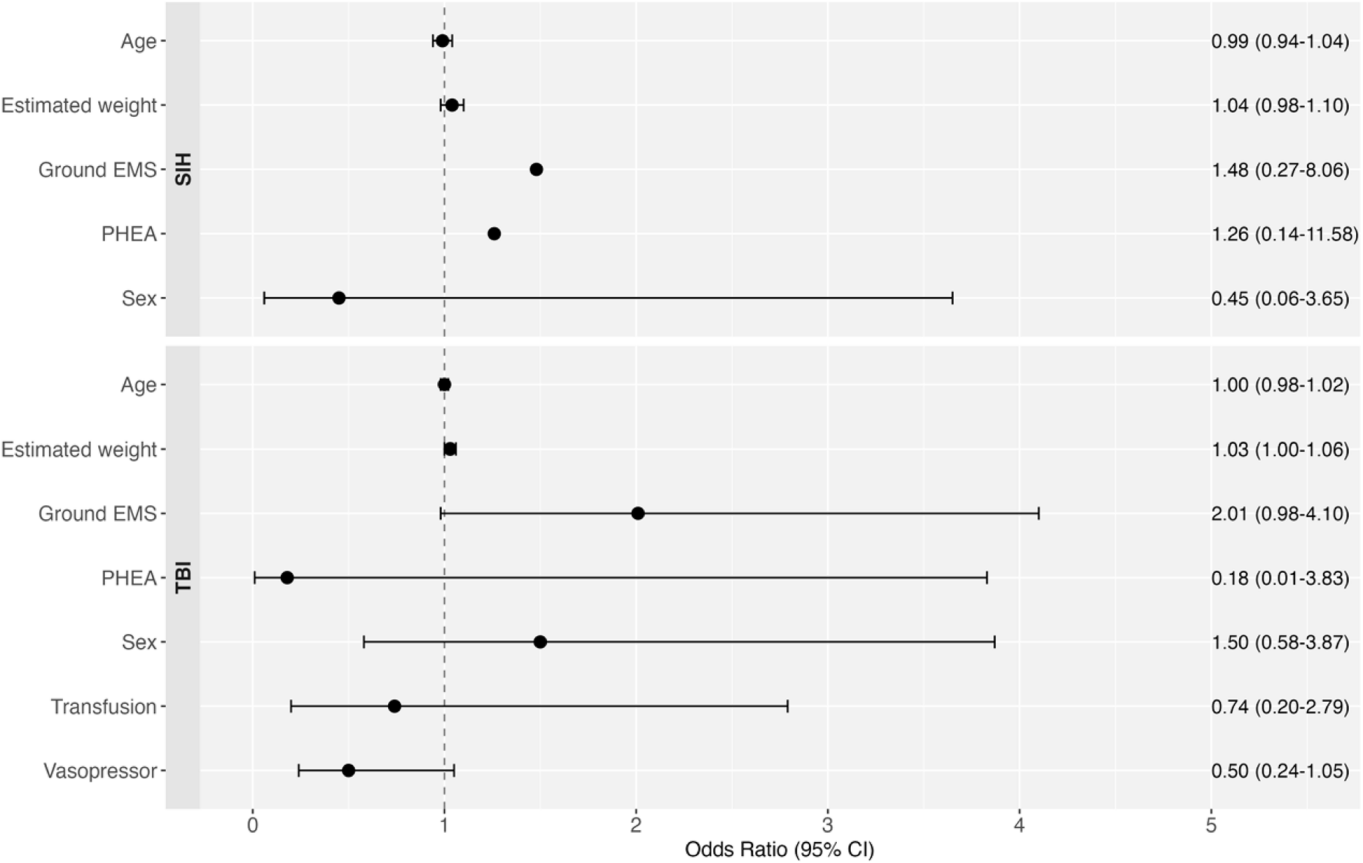
Forest plot of variables potentially associated with pairwise disagreement in mean arterial pressure between invasive and non-invasive blood pressure in patients with suspected TBI and sICH (n=209). TBI, traumatic brain injury; sICH, spontaneous intracranial haemorrhage; CI, confidence interval; EMS, emergency medical service; PHEA, pre-hospital emergency anaesthesia. NB: Sex denotes female sex as reference value; Ground EMS denotes conveyance type with aircraft as reference value

Univariate associations of patient variables with pairwise disagreement (MAP <10 mmHg) are presented in Additional file 1. In the multivariate mixed effects model only EMS conveyance of TBI patients was borderline associated with pairwise disagreement (OR 2.01, 95% CI 0.98–4.10, p = 0.05). In the sICH group, no fixed effects reached statistical significance (Fig. 2). The substantial random effects variance (1.61 for TBI and 1.92 for sICH) indicated considerable between-patient heterogeneity in MAP disagreement patterns. Forest plot of variables associated with pairwise disagreement in SBP and DBP are presented in Additional file 2.

### Relation between absolute blood pressure and pairwise disagreement

**Fig. 3:**
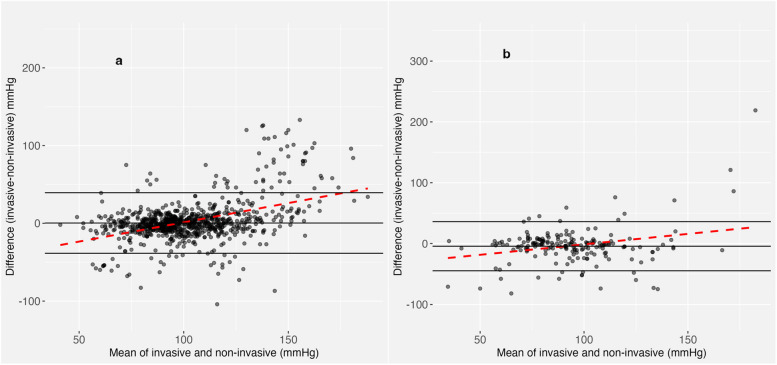
Bland-Altman plot of the difference between invasive and non-invasive mean arterial pressure measurements in patients with TBI (**a**) and sICH (**b**). Bland-Altman plots representing the mean difference in MAP (middle black horizontal line), the 95% limits of agreement (black solid lines) and bias (red dashed line) in TBI (**a**) and sICH (**b**)

To explore the relationship between absolute blood pressures and pairwise disagreement, Bland-Altman analysis with repeated measures was performed. In patients with TBI, the mean difference in MAP (accounting for the direction of the difference) between IBP and NIBP was 2.5 mmHg (limits of agreement −49.3 to 54.4 mmHg). A total of 66 concurrent measurements in 24 patients (6.4% of measurements, 15% of population) fell outside the limits of agreement. NIBP *over*estimated MAP in hypotensive patients, whereas it *under*estimated MAP in hypertensive patients (Fig. 3a). In patients with sICH, findings were similar (mean difference −3.5 mmHg, limits of agreement 56.4 to 63.4 mmHg) (Fig. 3b). Bland-Altman plots for SBP and DBP are shown in Additional file 3.

### Risk of pairwise disagreement

**Fig. 4:**
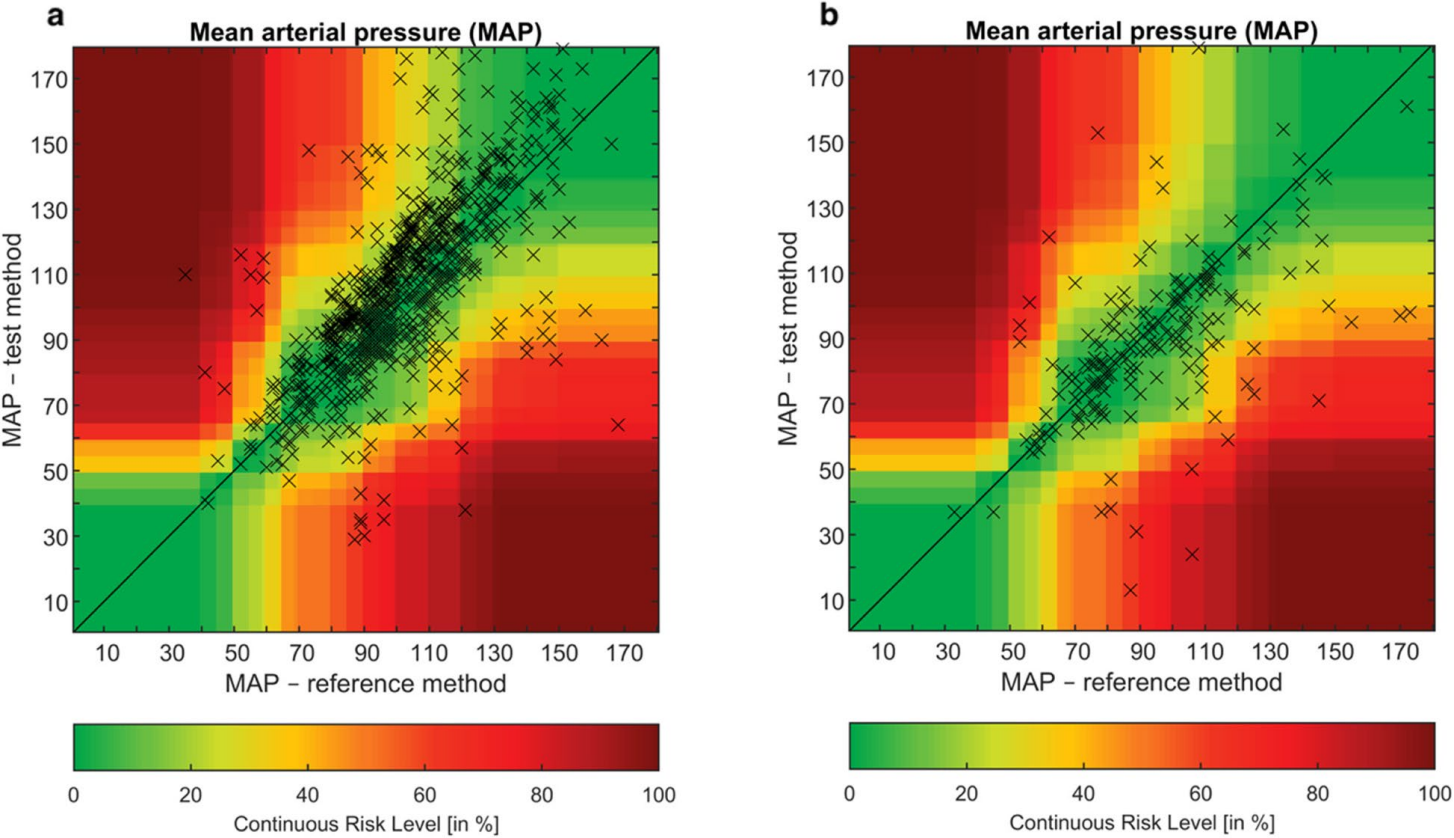
Error grid analysis for mean arterial pressure comparing non-invasive and invasive measurements in patients with suspected TBI (**a**) and sICH (**b**)**. a** Error Grid Analysis for TBI MAP (mmHg). **b** Error Grid Analysis for sICH MAP (mmHg). Invasive pressure (x-axis, reference standard) is plotted against non-invasive pressure (y-axis, index method). Data pairs marked by a black cross represent a concurrent data pair. Colour differentiation indicates the continuous risk level from the green (zone A, no risk) to dark red zone (zone E, dangerous risk). Zones A-E indicate: (A) no risk, no difference in clinical action, (B) low risk, benign or no treatment, (C) moderate risk, unnecessary treatment with moderate non-life-threatening consequences, (D) significant risk, unnecessary treatment with severe life-threatening consequences, (E) high risk, unnecessary treatment with severe life-threatening consequences. MAP; mean arterial pressure; TBI, traumatic brain injury; sICH, spontaneous intracranial haemorrhage

EGA for MAP in patients with TBI classified 69% (95% CI: 65.8–72.2), 24% (95% CI: 21.0–26.9), 4.3% (95% CI: 2.9–5.6), 1.5% (95% CI: 0.6–2.3), and 0.36% (95% CI: 0.0–0.7) of measurements in the risk categories A-E, respectively (Fig. 4). Based on this analysis, in 6.1% (95% CI: 4.4–7.7) of paired measurements NIBP differed from IBP with high clinical relevance (moderate to dangerous risk, categories C-E). For sICH, 12.5% (95% CI: 7.8–17.2) of paired measurements NIBP differed from IBP with high clinical relevance (moderate to dangerous risk, C-E). The cumulative percentage of MAP measurements falling within the low-risk categories (A-B) was 87.5% (95% CI: 82.8–92.2). EGA for SBP is available in Additional file 4.

## Discussion

Our findings suggest that when NIBP is used to guide pre-hospital haemodynamic optimisation in patients with TBI or sICH, clinically relevant measurement inaccuracies are observed in a significant proportion of patients, especially when hypo- or hypertension is present. To our knowledge, this is the first study to explore the use of pre-hospital IBP specifically in patients with a suspected brain insult. We suggest that pre-hospital invasive blood pressure monitoring has the potential to improve earlier haemodynamic optimisation, especially when hypo- or hypertension is present, further enabling tailored neuroprotection in the hyperacute phase.

As in previous (in-hospital) studies [18] the overall difference in average MAP between invasive- and non-invasive measurements in this study was small (-1.4 mmHg), and in accordance with thresholds set by the Association for the Advancement of Medical Instrumentation (AAMI) [19]. However, individual patient risk is not well expressed by this number, as the percentage of patients meeting criteria for pairwise disagreement between NIBP and IBP was substantial (42.6% of MAP values, 23% of SBP values and 34.4% of DBP values), reflecting a significant inter-individual variation, and potential for over- or undertreatment in a significant proportion of patients.

Pairwise disagreement was highest for MAP, and lowest for SBP. Differential reliability between blood pressure components is noteworthy given the measurement principles of modern pre-hospital monitors. These devices use oscillometric technology where MAP is directly measured at the point of maximum oscillations, while systolic and diastolic pressures are calculated using proprietary algorithms based on fixed ratios of oscillation amplitude [20]. MAP showed greater discrepancy, suggesting that oscillometric detection is vulnerable to motion artefacts during ambulance transport, while algorithmic SBP derivation appears more robust [20]. This is an important finding, as MAP is the main determinant of cerebral perfusion pressure (and thereby cerebral blood flow) [21, 22].

Of importance, pairwise agreement was related to absolute blood pressure values. Bland-Altman analysis demonstrated that NIBP *over*estimates low blood pressures and *under*estimates high blood pressures. The diverging pattern of measurements at clinical extremes and the wide limits of agreement suggest that non-invasive MAP measurements should be interpreted with caution, particularly when making clinical decisions in patients with very low or very high blood pressures. This is a critical finding as *under*estimation of blood pressure by NIBP, particularly at higher blood pressures, could lead to inadequate management of hypertension in sICH (and less so in TBI) patients. Conversely, *over*estimation at lower ranges might result in delayed recognition and treatment of hypotension, further exacerbating secondary brain injury [23]. Although the deleterious effects of hypertension in TBI are less well established [22], early hypotension has been associated with a poor outcome [21].

Error grid analysis demonstrated that the above mentioned under- and overestimations are of clinical relevance. For TBI patients, 6.1% of the MAP measurements fell in the ‘moderate to dangerous risk’ categories, indicating that measurement inaccuracies are of a magnitude that they could result in harmful treatment decisions, or omission of treatment. For sICH patients this number was even as high as 12.5%. Although treatment decisions are generally made based on physiological trends rather than single measurements, our findings suggest that at any given time-point non-invasive monitoring may potentially alter therapeutic decision-making in a significant proportion of patients, thereby affecting the efficacy of pre-hospital neuroprotective care [11, 24–26]. Furthermore, over 90% of the TBI patients in our study required PHEA. Peri-PHEA NIBP measurement accuracy is important not only to deliver a timely, safe and effective emergency anaesthetic [27], but also to establish post-PHEA maintenance of anaesthesia through infusion therapies and appropriate vasopressor dosages [28, 29].

Pre-hospital IBP monitoring is critical for maintaining haemodynamic optimisation in TBI or sICH patients receiving PHEA, especially during extended transport intervals. Despite minor scene delays from arterial line insertion [24, 30], the substantial clinical benefits strongly justify this approach before PHEA administration. In our cohort, 153/198 (77.3%) patients received arterial access pre-PHEA, demonstrating concordance with established in-hospital anaesthesia guidelines [31, 32]. Pre-hospital invasive monitoring does present challenges, including vascular complications and damping phenomena that can compromise treatment decisions [33]. Clinicians must carefully weigh these limitations against the significant advantages of precise blood pressure management when caring for neurologically injured patients in the pre-hospital environment. Further, arterial line monitoring may facilitate serial blood gas analysis allowing for precise ventilation management [34], more accurate titration of vasopressors, titration of post PHEA anaesthesia and arterial waveform analysis. These extend the benefit of arterial monitoring beyond haemodynamic optimisation, to broader neuroprotection strategies.

Our study has several limitations. First, the retrospective design limits our ability to control for potential confounding factors and (selection) bias. For example, the average measurements per patient was 9 (IQR 3–15, range: 1–29) with a ‘pair’ defined anywhere within a 60 second time-interval. Although the frequency largely represents clinical practice dependant upon clinical need and transport duration, it does mean that patients with more measurements would contribute disproportionately to the analysis. Second, for 100,006 IBP data pairs, no concurrent NIBP measurement was available. Although this could largely be attributed to the higher sampling frequency, in some patients NIBP was likely discontinued when IBP measurements were available, with potential for systematic bias. Third, in some patients NIBP may not have been recorded as they were in a critically hypotensive- or hypertensive state. Although excluding these patients may have resulted in a selection bias, it is an additional argument to support pre-hospital invasive blood pressure monitoring. Fourth, although we attempted to exclude artefactual measurements, the dynamic pre-hospital environment may introduce measurement errors that are difficult to eliminate in retrospect. Despite following established protocols for arterial line setup and maintenance, we cannot retrospectively quantify the prevalence or impact of subtle waveform damping that may have influenced IBP measurements. While grossly abnormal waveforms would have been identified and corrected by clinicians, minor damping effects could still have affected absolute pressure values without being detected. This limitation is inherent to retrospective analysis of arterial pressure monitoring data. Future prospective studies should include systematic documentation of damping coefficients to further characterise measurement inaccuracy. Fifth, a limitation specific to EGA pertains to the geometric shape and zonal placement within the error grid. Marginally different arterial pressure measurements may be assigned to different zones termed ‘zone skipping’ [18]. Further, error grids are derived from a survey among twenty-five experts in the field of anaesthesiology and intensive care medicine lacking response validation, therefore the zones are based on perceived and not real-time clinical action and may only *predict* the clinical relevance of discrepancy in this cohort. Finally, the single-centre design limits generalisability to other systems with varying resources, geographical challenges and standard operating procedures. In our cohort, median GCS was 6, and 94.7% received PHEA, therefore the merit of IBP may prove less in patients with greater haemodynamic stability.

## Conclusion

Pre-hospital non-invasive blood pressure to guide haemodynamic optimisation of patients with TBI or sICH is hampered by clinically relevant measurement inaccuracies in a significant proportion of patients. We find that pre-hospital invasive blood pressure monitoring has the potential to improve early haemodynamic optimisation, especially when hypo- or hypertension is present, supporting one component of tailored neuroprotection in the hyperacute phase.

## Supplementary Information

Additional file 1. Univariate and multivariate analysis of variables associated with pairwise disagreement in mean arterial pressure (>10%), systolic blood pressure (>20%) and diastolic blood pressure (>20%) in in patients with suspected TBI (n=159) and sICH (n=50).

Additional file 2. Forest plot of variables potentially associated with a SBP discrepancy > 20mmHg (Fig. 2a) and a DBP discrepancy > 20 mmHg (Fig. 2b) between invasive- and non-invasive monitoring in patients with suspected TBI (n=159) and sICH (n=50).

Additional file 3. Bland-Altman plot of the difference between invasive and non-invasive systolic- and diastolic blood pressure measurements in patients with suspected TBI (Fig. 3a and b) and sICH (Fig. 3c and d).

Additional file 4. Error grid analysis for systolic blood pressure comparing non-invasive and invasive measurements in patients with suspected TBI (Fig. 4a) and sICH (Fig. 4b).

## Data Availability

No datasets were generated or analysed during the current study.

## Abbreviations

AAGBI: Association of Anaesthetists of Great Britain and Ireland
CI: Confidence Interval
DBP: Diastolic Blood Pressure
EGA: Error Grid Analysis
EMS: Emergency Medical Services
GCS: Glasgow Coma Scale
HEMS: Helicopter Emergency Medical Services
HRA: Health Research Authority
IBP: Intra-arterial Blood Pressure
IQR: Interquartile Range
LA: Limits of Agreement
MAP: Mean Arterial Pressure
MTC: Major Trauma Centre
NIBP: Non-invasive Blood Pressure
NIHR: National Institute of Health and Care Research
iDBP: Invasive Diastolic Blood Pressure
iSBP: Invasive Systolic Blood Pressure
KSS: Air Ambulance Charity Kent Surrey Sussex
OR: Odds Ratio
RCoA: Royal College of Anaesthetists
SBP: Systolic Blood Pressure
sICH: Spontaneous Intracranial Haemorrhage
STROBE: Strengthening the Reporting of Observational Studies in Epidemiology
TBI: Traumatic Brain Injury
