# Supplementary material for: Diagnostic accuracy of pre-hospital invasive arterial blood pressure monitoring for haemodynamic management in traumatic brain injury and spontaneous intracranial haemorrhage
Source: Scand J Trauma Resusc Emerg Med. 2025 May 16;33:89. doi: 10.1186/s13049-025-01393-4 (PMC12082994; doi:10.1186/s13049-025-01393-4)
Supplement: Supplementary file 1 — Additional file 1. Univariate and multivariate analysis of variables associated with pairwise disagreement in mean arterial pressure (>10%), systolic blood pressure (>20%) and diastolic blood pressure (>20%) in in patients with suspected TBI (n=159) and sICH (n=50). [file 13049_2025_1393_MOESM1_ESM.docx]

**Additional file 1**

**Univariate and multivariate analysis of variables associated with pairwise disagreement in mean arterial pressure (>10%), systolic blood pressure (>20%) and diastolic blood pressure (>20%) in in patients with suspected TBI (n=159) and sICH (n=50).**

|  |  | **TBI Univariate** | | **TBI Multivariate** | | **sICH Univariate** | | **sICH Multivariate** | |
| --- | --- | --- | --- | --- | --- | --- | --- | --- | --- |
| **BP Type** | **Variable** | **OR (95% CI)** | **p-value** | **OR (95% CI)** | **p-value** | **OR (95% CI)** | **p-value** | **OR (95% CI)** | **p-value** |
| MAP | Age | 1.00 (0.98-1.02) | 0.70 | 0.99 (0.95-1.02) | 0.58 | 0.99 (0.94-1.04) | 0.64 | 1.00 (0.98-1.02) | 0.85 |
|  | Sex | 1.14 (0.46-2.82) | 0.78 | 0.45 (0.06-3.65) | 0.45 | 0.45 (0.06-3.65) | 0.46 | 1.50 (0.58-3.87) | 0.39 |
|  | Estimated weight | 1.02 (0.99-1.05) | 0.23 | 1.04 (0.98-1.10) | 0.21 | 1.04 (0.98-1.10) | 0.21 | 1.03 (1.00-1.06) | 0.08 |
|  | PHEA | 0.29 (0.02-4.33) | 0.41 | 1.26 (0.14-11.58) | 0.84 | 1.26 (0.14-11.58) | 0.84 | 0.18 (0.01-3.63) | 0.26 |
|  | Transfusion | 0.64 (0.16-2.48) | 0.51 | - | - | - | - | 0.74 (0.20-2.79) | 0.66 |
|  | Vasopressor | 0.62 (0.29-1.29) | 0.19 | - | - | - | - | 0.50 (0.24-1.05) | 0.07 |
|  | Ground EMS | 1.98 (0.97-4.05) | 0.05 | 1.48 (0.27-8.06) | 0.65 | 1.48 (0.27-8.06) | 0.65 | 2.01 (0.98-4.10) | 0.06 |
| SBP | Age | 1.01 (0.99-1.02) | 0.42 | 1.00 (0.94-1.04) | 0.64 | 1.00 (0.97-1.03) | 0.64 | 1.01 (0.99-1.02) | 0.41 |
|  | Sex | 1.03 (0.43-2.49) | 0.94 | 0.98 (0.15-6.37) | 0.46 | 0.37 (0.11-1.22) | 0.10 | 1.03 (0.43-2.49) | 0.94 |
|  | Estimated weight | 1.01 (0.98-1.04) | 0.54 | 1.00 (0.95-1.05) | 0.21 | 1.00 (0.97-1.03) | 0.93 | 1.01 (0.98-1.04) | 0.54 |
|  | PHEA | 0.37 (0.02-5.93) | 0.48 | 0.39 (0.15-1.02) | 0.05 | 0.38 (0.14-1.02) | 0.05 | 0.37 (0.02-5.93) | 0.48 |
|  | Transfusion | 1.43 (0.41-4.97) | 0.58 | - | - | - | - | 1.43 (0.41-4.97) | 0.58 |
|  | Vasopressor | 0.89 (0.45-1.78) | 0.75 | - | - | - | - | 0.89 (0.45-1.78) | 0.75 |
|  | Ground EMS | 2.73 (1.29-5.78) | 0.01* | 1.81 (0.73-4.48) | 0.20 | 1.81 (0.73-4.48) | 0.20 | 2.73 (1.29-5.78) | 0.01* |
| DBP | Age | 1.00 (0.98-1.02) | 0.85 | 0.96 (0.92-1.01) | 0.12 | 0.96 (0.92-1.01) | 0.12 | 1.00 (0.98-1.02) | 0.85 |
|  | Sex | 1.38 (0.52-3.67) | 0.52 | 0.98 (0.15-6.37) | 0.99 | 0.98 (0.15-6.37) | 0.99 | 1.38 (0.52-3.67) | 0.52 |
|  | Estimated weight | 1.05 (1.01-1.09) | 0.01* | 1.00 (0.95-1.05) | 0.93 | 1.00 (0.95-1.05) | 0.93 | 1.05 (1.01-1.09) | 0.01* |
|  | PHEA | 0.12 (0.01-2.28) | 0.16 | 3.90 (0.48-31.54) | 0.20 | 3.90 (0.48-31.54) | 0.20 | 0.12 (0.01-2.28) | 0.16 |
|  | Transfusion | 0.74 (0.19-2.94) | 0.67 | - | - | - | - | 0.74 (0.19-2.94) | 0.67 |
|  | Vasopressor | 0.58 (0.27-1.26) | 0.17 | - | - | - | - | 0.58 (0.27-1.26) | 0.17 |
|  | Ground EMS | 2.73 (1.29-5.78) | 0.01* | 1.48 (0.31-6.94) | 0.62 | 1.48 (0.31-6.94) | 0.62 | 2.73 (1.29-5.78) | 0.01* |

**Legend.** Univariate and multivariate analysis of predictors with blood pressure disagreement. Pre-defined blood pressure disagreement was regarded as SBP/DBP >20 mmHg and MAP >10mmHg. TBI, traumatic brain injury; sICH, spontaneous traumatic haemorrhage; MAP, mean arterial pressure; SBP, systolic blood pressure; DBP, diastolic blood pressure; OR, odds ratio; CI, confidence interval. Significance level * p< 0.05.
