# Supplementary material for: Diagnostic accuracy of pre-hospital invasive arterial blood pressure monitoring for haemodynamic management in traumatic brain injury and spontaneous intracranial haemorrhage
Source: Scand J Trauma Resusc Emerg Med. 2025 May 16;33:89. doi: 10.1186/s13049-025-01393-4 (PMC12082994; doi:10.1186/s13049-025-01393-4)
Supplement: Supplementary file 2 — Additional file 2. Forest plot of variables potentially associated with a SBP discrepancy > 20mmHg (Fig. 2a) and a DBP discrepancy > 20 mmHg (Fig. 2b) between invasive- and non-invasive monitoring in patients with suspected TBI (n=159) and sICH (n=50). [file 13049_2025_1393_MOESM2_ESM.docx]

**Additional file 2**

**Forest plot of variables potentially associated with a SBP discrepancy > 20mmHg (Figure 2a) and a DBP discrepancy > 20 mmHg (Figure 2b) between invasive- and non-invasive monitoring in patients with suspected TBI (n=159) and sICH (n=50).**

**
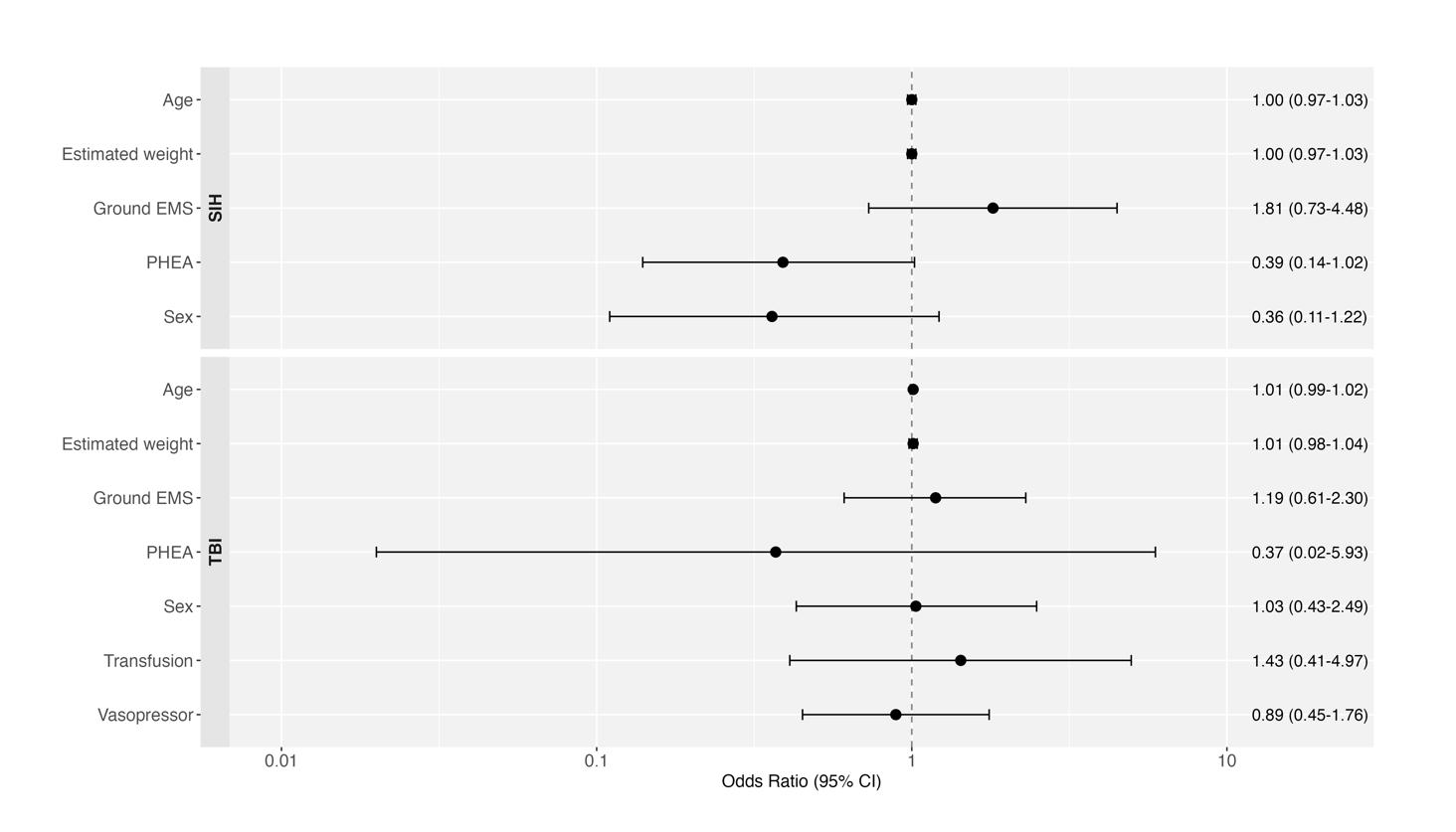
**

**Legend.** Multivariate analysis of predictors with SBP disagreement. Pre-defined blood pressure disagreement was regarded as SBP >20 mmHg. TBI, traumatic brain injury; sICH, spontaneous traumatic haemorrhage; EMS, Emergency Medical Service; PHEA, Pre-hospital Emergency Anaesthesia; OR, odds ratio; CI, confidence interval.

**
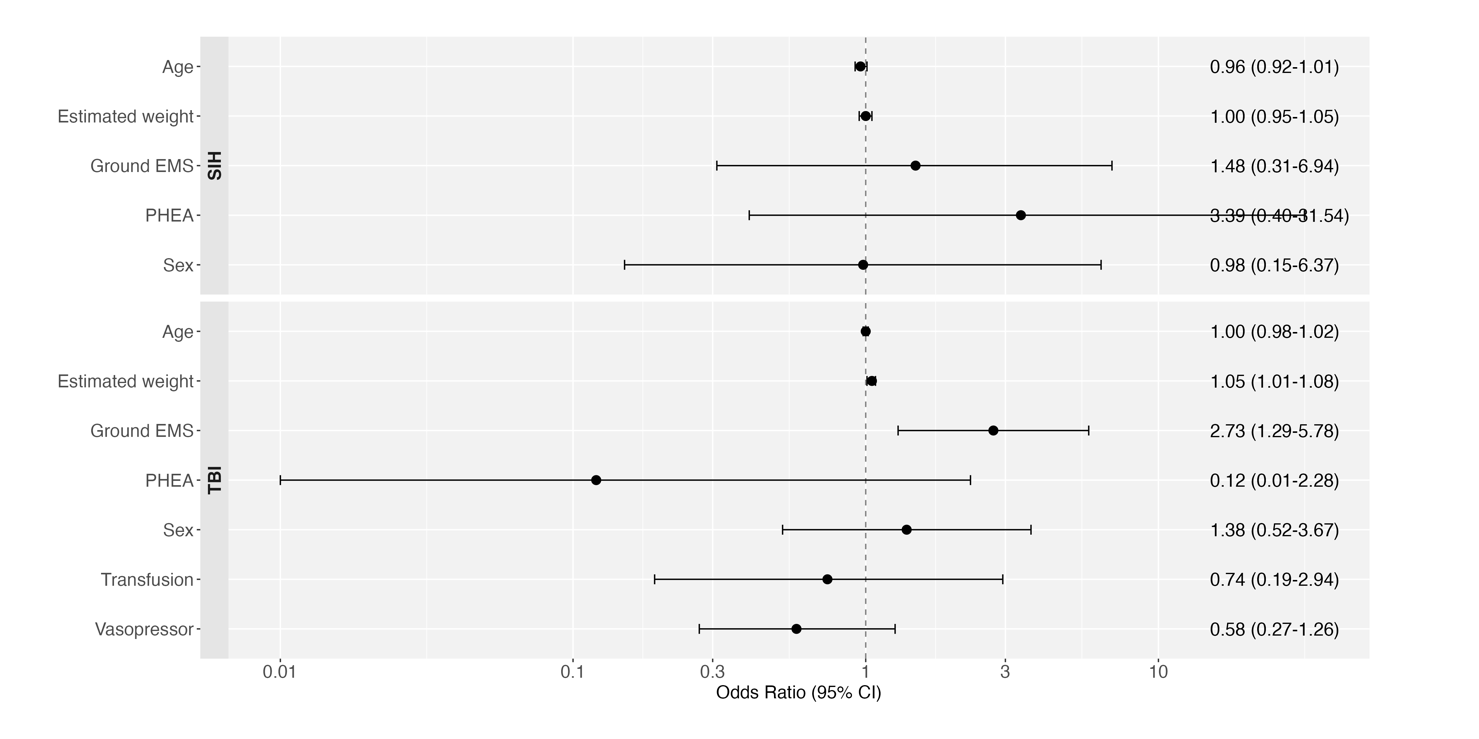
**

**Legend.** Multivariate analysis of predictors with DBP disagreement. Pre-defined blood pressure disagreement was regarded as DBP >20 mmHg. TBI, traumatic brain injury; sICH, spontaneous traumatic haemorrhage; EMS, Emergency Medical Services; PHEA, Pre-hospital Emergency Anaesthesia; OR, odds ratio; CI, confidence interval. Significance level * p< 0.05, ** p< 0.01, *** p< 0.001.
