# Supplementary material for: Diagnostic accuracy of pre-hospital invasive arterial blood pressure monitoring for haemodynamic management in traumatic brain injury and spontaneous intracranial haemorrhage
Source: Scand J Trauma Resusc Emerg Med. 2025 May 16;33:89. doi: 10.1186/s13049-025-01393-4 (PMC12082994; doi:10.1186/s13049-025-01393-4)
Supplement: Supplementary file 3 — Additional file 3. Bland-Altman plot of the difference between invasive and non-invasive systolic- and diastolic blood pressure measurements in patients with suspected TBI (Fig. 3a and b) and sICH (Fig. 3c and d). [file 13049_2025_1393_MOESM3_ESM.docx]

**Additional file 3**

**
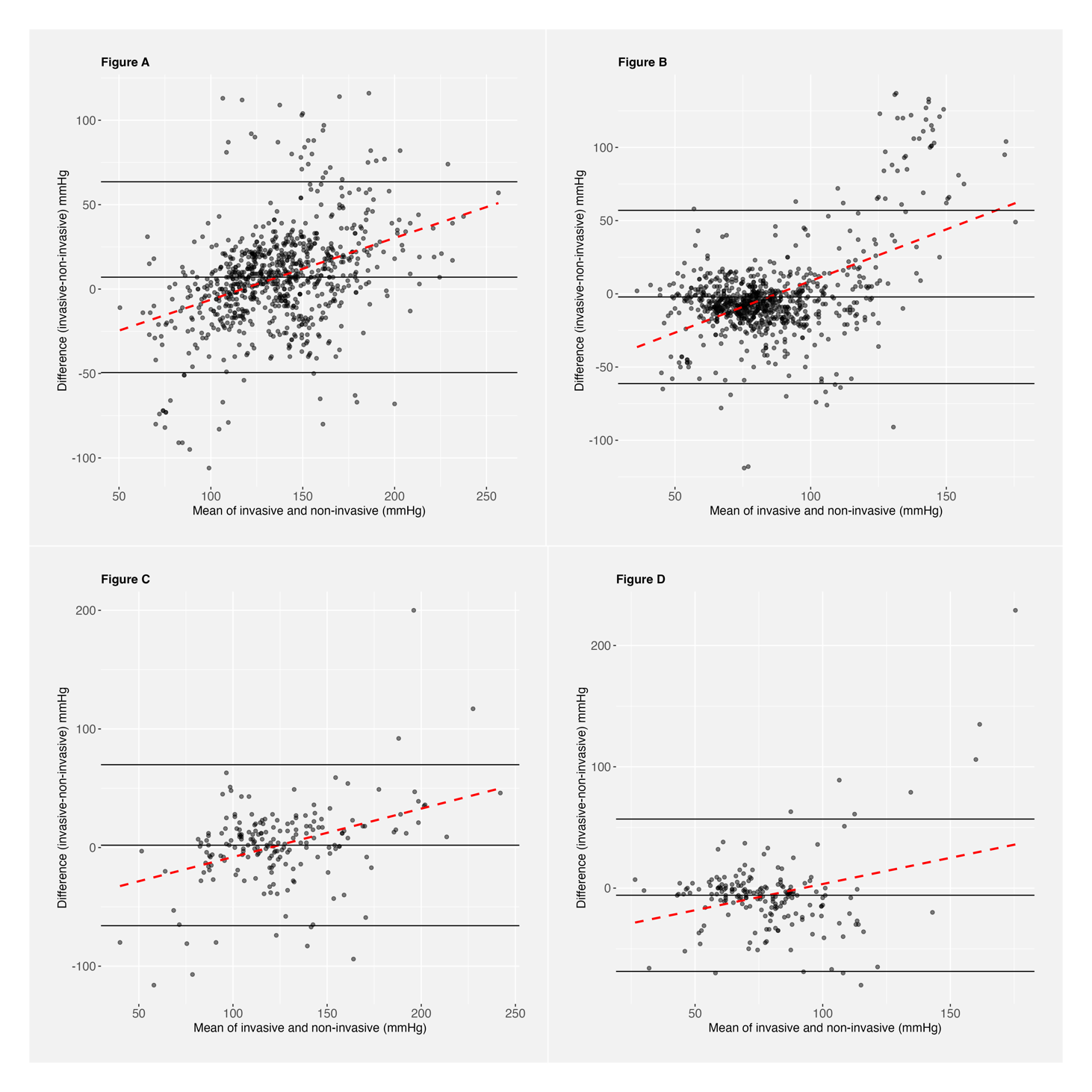
Bland-Altman plot of the difference between invasive and non-invasive systolic- and diastolic blood pressure measurements in patients with suspected TBI (Figure 3a and 3b) and sICH (Figure 3c and 3d).**

**Legend figure 3a.** Bland-Altman plot for the mean difference in SBP (black horizontal line), the 95% limits of agreement (black solid lines) and bias (red dashed line) in the TBI cohort. Mean difference 7.05mmHg, Standard deviation of differences 28.83mmHg, Upper Limit of Agreement 63.56mmHg, Lower Limit of Agreement -49.45mmHg. **Figure 3b.** Bland-Altman plot for the mean difference in DBP (black horizontal line), the 95% limits of agreement (black solid lines) and bias (red dashed line) in the TBI cohort. Mean difference -2.15mmHg, Standard deviation of differences 30.18mmHg, Upper Limit of Agreement 57.01mmHg, Lower Limit of Agreement -61.31mmHg. **Figure 3c.** Bland-Altman plot for the mean difference in SBP (black horizontal line), the 95% limits of agreement (black solid lines) and bias (red dashed line) in the sICH cohort. Mean difference 1.99mmHg, Standard deviation of differences 34.62mmHg, Upper Limit of Agreement 69.85mmHg, Lower Limit of Agreement -65.86mmHg. **Figure 3d.** Bland-Altman plot for the mean difference in DBP (black horizontal line), the 95% limits of agreement (black solid lines) and bias (red dashed line) in the SIH cohort. Mean difference -5.87mmHg, Standard deviation of differences 32.04mmHg, Upper Limit of Agreement 56.92mmHg, Lower Limit of Agreement -68.66mmHg. SBP, systolic blood pressure. DBP, diastolic blood pressure. TBI, traumatic brain injury. sICH, spontaneous intracerebral haemorrhage.
