# Supplementary material for: Diagnostic accuracy of pre-hospital invasive arterial blood pressure monitoring for haemodynamic management in traumatic brain injury and spontaneous intracranial haemorrhage
Source: Scand J Trauma Resusc Emerg Med. 2025 May 16;33:89. doi: 10.1186/s13049-025-01393-4 (PMC12082994; doi:10.1186/s13049-025-01393-4)
Supplement: Supplementary file 4 — Additional file 4. Error grid analysis for systolic blood pressure comparing non-invasive and invasive measurements in patients with suspected TBI (Fig. 4a) and sICH (Fig. 4b). [file 13049_2025_1393_MOESM4_ESM.docx]

**Additional file 4**

**Error grid analysis for systolic blood pressure comparing non-invasive and invasive measurements in patients with suspected TBI (Figure 4a) and sICH (Figure 4b).**

**
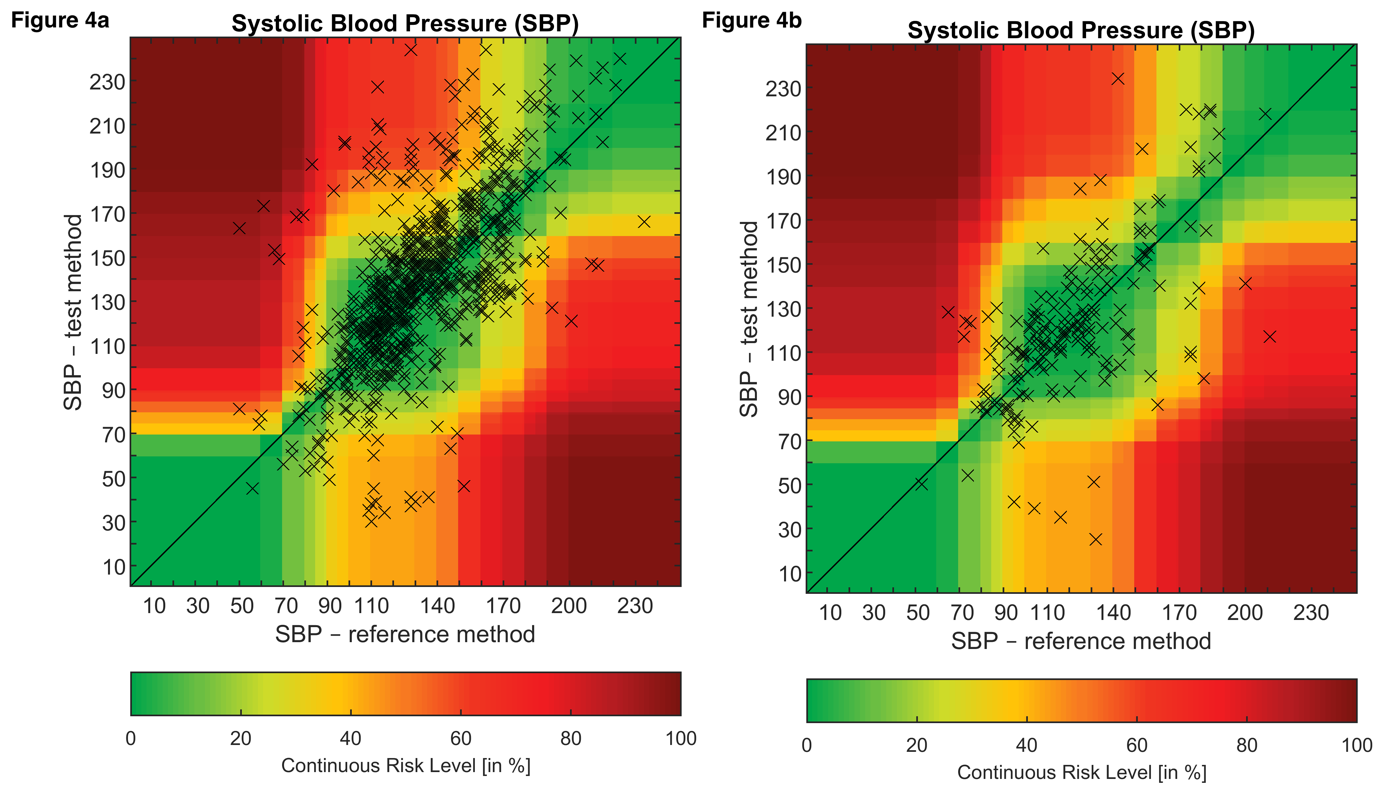
**

**Legend figure 4a.** EGA for SBP in TBI classified 66% (95% CI: 62.71-69.29), 24% (95% CI: 21.07-26.93), 8.9% (95% CI: 6.98-10.82), 0.84% (95% CI: 0.22-1.46), and 0.60% (95% CI: 0.08 - 1.12) of measurements in the risk categories A-E, respectively (Figure 1). Based on this analysis, in 10.3% (95% CI: 8.28-12.32) of paired measurements NIBP differed from IBP with high clinical relevance (moderate to dangerous risk, C-E). The cumulative percentage of SBP measurements falling within the low-risk categories (A-B) was 90% (95% CI: 87.68-92.32). **Figure 4b.** EGA for SBP in SIH classified 64.2% (95% CI: 57.4-71.0), 22.3% (95% CI: 16.4-28.2), 9.8% (95% CI: 5.6-14.0), 3.1% (95% CI: 0.7-5.5), and 0.5% (95% CI: 0.0-1.2) of measurements in the risk categories A-E, respectively. Based on this analysis, in 13.4% (95% CI: 8.6-18.2) of paired measurements NIBP differed from IBP with high clinical relevance (moderate to dangerous risk, C-E). The cumulative percentage of SBP measurements falling within the low-risk categories (A-B) was 86.6% (95% CI: 81.8-91.4).
